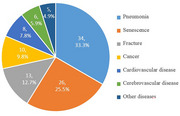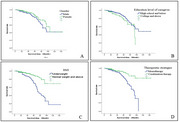# Survival analysis of patients with Alzheimer's disease in a memory clinic in Xi'an, China

**DOI:** 10.1002/alz70860_099200

**Published:** 2025-12-23

**Authors:** Xiaojuan Guo, Jie Liu, Ling Gao, Wenhui Lu, Qiumin Qu

**Affiliations:** ^1^ The First Affiliated Hospital of Xi’an Jiaotong University, Xi’an, Shaanxi, China; ^2^ The First Affiliated Hospital of Xi'an Jiaotong University, Xi’an, Shaanxi, China; ^3^ The First Affiliated Hospital of Xi'an Jiaotong University, Xi'an, Shaanxi, China

## Abstract

**Background:**

The survival status of patients with Alzheimer's disease (AD) in China has not been determined. This paper aimed to explore the survival status of AD patients and evaluate the potential determinants of survival in Xi'an, Northwest China.

**Method:**

AD patients from the memory clinic of the first affiliated hospital of Xi’an Jiaotong university were evaluated at baseline and followed up regularly. Survival analysis was performed using the Kaplan‐Meier method, and a Cox proportional hazard model was used to assess factors associated with patient survival.

**Result:**

There were 617 AD patients included in the analysis. During the follow‐up, 102 patients died. The age at death was 77.0 ± 7.6 years, and the survival time after onset of the disease was 8.9 ± 0.3 years. Pneumonia (33.3%) and senescence (25.5%) were the most common causes for the death of AD patients. The factors associated with patient survival included diagnostic delay (HR = 2.45, 95% CI: 1.59‐3.78, *p* < 0.001), fall accident (HR = 1.56, 95% CI: 1.04‐2.35, *p* = 0.031), body mass index (HR = 0.61, 95% CI: 0.40‐0.92, *p* = 0.020), occupation of caregiver (HR = 0.58, 95% CI: 0.39‐0.87, *p* = 0.008), therapeutic strategies (HR = 0.52, 95% CI: 0.30‐0.89, *p* = 0.017) and medication compliance (HR = 0.37, 95% CI: 0.22‐0.59, *p* < 0.001).

**Conclusion:**

The mean survival time of AD patients in Xi'an, Northwest China was 8.9 years. Pneumonia was the leading cause of death for AD patients. Diagnostic delay and fall accident were associated with shorter survival, while good nutrition, caregivers and medication therapy were associated with good outcome.